# Flowcytometric data of intermediate-large cell gastrointestinal lymphoma presenting a gross mass in 32 cats – “let them glow in the flow”

**DOI:** 10.3389/fvets.2024.1378826

**Published:** 2024-05-28

**Authors:** Barbara C. Rütgen, Birgitt Wolfesberger, Daniel Baumgartner, Sabine E. Hammer, Sandra Groiss, Katharina M. Hittmair, Gabriele Gradner, Andrea Fuchs-Baumgartinger, Taryn A. Donovan, Ilse Schwendenwein

**Affiliations:** ^1^Clinical Pathology, Department of Biological Sciences and Pathobiology, University of Veterinary Medicine Vienna, Vienna, Austria; ^2^Clinical Unit of Internal Medicine Small Animals, Department for Companion Animals and Horses, University of Veterinary Medicine Vienna, Vienna, Austria; ^3^Institute of Immunology, Department of Biological Sciences and Pathobiology, University of Veterinary Medicine Vienna, Vienna, Austria; ^4^Diagnostic Imaging, Department for Companion Animals and Horses, University of Veterinary Medicine Vienna, Vienna, Austria; ^5^Small Animal Hospital Clinic, Department for Companion Animals and Horses, University of Veterinary Medicine Vienna, Vienna, Austria; ^6^Institute of Pathology, Department of Biological Sciences and Pathobiology, University of Veterinary Medicine Vienna, Vienna, Austria; ^7^The Schwarzman Animal Medical Center, New York, NY, United States

**Keywords:** feline lymphoma, flow cytometry, immunophenotyping, gastrointestinal, WHO classification

## Abstract

Gastrointestinal lymphoma is the most common form of lymphoma in domestic cats. Aggressive phenotypes are much less common but do bear and unfavorable prognosis. Immunophenotyping by flow cytometry (FCM) is not systematically performed in these patients, because of difficulties in the acquisition of suitable sample material from the gastrointestinal tract. A multimodal diagnostic approach is recommended to improve identification of subtypes targeting patient tailored therapeutic strategies. The aim of this prospective study was to present results of multicolor FCM immunophenotyping in surgically removed gastrointestinal mass and relate them with histopathology using the World Health Organization (WHO) classification and clonality PCR testing. Thirty-two patients were included. Eight cats (25%) had gastric, 23 (72%) had intestinal lymphoma and 1 (3%) had gastric/jejunal lymphoma. Intestinal lymphoma sites were represented by 18 small intestinal, 4 ileocaecal, 1 large intestinal. All gastric lymphomas were diffuse large B-cell lymphoma (DLBCL). Small intestinal lymphomas were 10 enteropathy associated T-cell lymphoma type I (EATL I), 2 enteropathy associated T-cell lymphoma type II (EATL II), 2 peripheral T-cell lymphoma (PTCL), 3 DLBCL and one DLBCL+EATL II. The most common small intestinal FCM T-cell phenotype was CD3^+^CD21^−^ CD4^−^CD8^−^CD18^+^ CD5^−^CD79^−^ in 7/10 EATL I and one EATL II. The most frequent FCM B-cell phenotype was CD3^−^CD21^+^ CD4^−^CD8^−^CD18^+^ CD5^−^CD79^+^ in 13/17 DLBCL and the DLBCL+EATL II. Clonality PCR results were positive in 87.5% (28/32) of all cases. No cross-lineage rearrangement was observed. IHC and FCM results agreed in 87.5% (28/32) of all cases. When all 3 methods were combined, consistent results were seen in 75% (24/32). This is the first demonstration of a multicolor FCM approach set in context to the gold standard histopathology and clonality testing results.

## 1 Introduction

Lymphoma is a common neoplasia of domestic cats, affecting multiple organ systems (1). In felines the gastrointestinal tract is the most common anatomic site of manifestation (2). Low-grade small intestinal T-cell lymphoma was recently described in detail (3, 4), but aggressive subtypes bearing an unfavorable prognosis are also observed (5, 6).

Currently, the World health organization (WHO) histologic classification is the diagnostic gold standard for feline lymphoma (7, 8) requiring a full thickness biopsy specimen, which is invasive and time-consuming (9). In contrast to the dog, feline gastrointestinal lymphoma, with the stomach and small intestines as the most commonly affected gastrointestinal regions, is not routinely diagnosed with rapid tools such as flow cytometry (FCM). An important reason for this is that fine needle aspirates of sufficient quantity and quality for FCM are difficult to obtain from these sites, thus, FCM is not an integral part of diagnostic workup. For this reason, descriptions of immunophenotypic expression patterns including CD4CD8 expression are scarce.

However, the fact that feline antibodies against CD4 and CD8 are only available for FCM or frozen biopsy samples, and not for paraffin embedded material, makes this method interesting for gaining basic insights into expression patterns and to expand knowledge (3, 10).

So far four retrospective studies on feline lymphoma mentioned FCM analysis in intestinal tissue, however, no systematically collected data is available (9, 11–13).

Roccabianca et al. (11) used FCM for the first time in feline intestinal tissues characterizing the non-malignant intraepithelial compartment (IEC) and the lamina propria compartment (LPC) lymphocytes in the intestines of 22 specific pathogen free (SPF) cats. The most comprehensive retrospective overview on FCM in feline lymphoma elucidates pre-analytic factors affecting the sample quality of peripheral lymph nodes, as well as abdominal or thoracic lymph nodes and masses (9). Guzera et al. (12) retrospectively analyzed 19 FNA samples of lymphoid material and documented the immunophenotype. Thirteen lymphoma cases consisted of 6 alimentary lymphoma samples of which five were of B-cell and one of T-cell origin (12). The most recent publication reported FCM data of different anatomic sites including GI-tract but also spleen and tarsus of seven cats and six dogs (13). FCM was only used in one patient. In this recent publication, four-color staining was chosen. In the other aforementioned papers, a single- or two-color approach was used.

In summary FCM data characterizing feline intestinal lymphoma is based on 6 cases in a single color stain (12). There is no information available about provable eg CD4CD8 double expression or multicolor staining.

Herewith we present FCM results gained by a multi-color approach from thirty-two cats with GI-lymphoma. Data are presented in relation to WHO classification and PCR clonality testing obtained from identical sampling sites. Multicolor FCM might be useful for identifying different expression patterns of feline alimentary lymphoma, aiding potential future patient tailored therapeutic approaches.

## 2 Materials and methods

### 2.1 Patient selection

Inclusion criteria for this study were presence of a surgically removable lymphoma mass exclusively present in the gastrointestinal tract, which was confirmed during the staging process. Lymphoma cases with diffuse wall thickening were not included in this series.

An initial tentative diagnosis was obtained by cytologic evaluation of an ultrasound guided fine needle aspiration smear of a suspect tumor mass from cats that were presented to the Clinic for Small Animals at the University of Veterinary Medicine, Vienna, Austria, between April 2016 and January 2021. With informed owners consent for further diagnostic workup and therapy, sample material was obtained via surgical procedures in which gross gastrointestinal masses were removed completely.

Data about the 3D size of the material used for the study was documented in cm^3^.

The study was approved by an Ethics Committee and the Federal Ministry for Science and Research (referring number: GZ 68.205/0173-WF/V/3b/2015).

### 2.2 Patient material

Within 1 h of surgery, the excised neoplastic gastrointestinal material was processed. Immediately after excision the material was transferred into a 15 mL vial (Greiner Bio-One, Kremsmünster, Austria) containing 10 mL cell culture medium (RPMI, PAA, Pasching, Austria) supplemented with 10% inactivated fetal calf serum (FCS, PAA) and 100 U/mL penicillin/0.1 mg/mL streptomycin (PAA). The material was stored at 4°C for a maximum of 1 h until further processing, depending on the time of surgery. All tissue from each cat was then trimmed clean of surrounding fat and connective tissue and was cut in half. Tissue imprints were prepared for cytologic evaluation to affirm the presence of lymphoid cells in tumor material before immunophenotyping. Representative tumor mass was separately transferred into 4% buffered formaldehyde solution and prepared for histopathology and immunohistochemistry. From the neoplastic intestinal material in the cell culture medium, a single cell suspension was prepared by mincing the tissue through a sieve (mesh size 40 μm) and flushing bigger tissue parts with a syringe (BD Micro-Fine 0.5 mL Insulin syringe U-100, New Jersey, United States) using phosphate buffered saline (1 × PBS) without Ca2+ and Mg2+ (PAA). The single cell suspension was transferred to phosphate buffered saline and was centrifuged at 1300 U/min (353 g) for 6 min (Heraeus Multifuge 1 s-R, Kendro, Osterode, Germany). The cell pellet was resuspended in 1 mL 1 × PBS and was used for Flow Cytometric immunophenotyping and PCR-based lymphocyte clonality testing.

### 2.3 Total nucleated cell count of single cell suspension for FCM

The total nucleated cell count (TNCC) was determined by an ADVIA 2120™ (Siemens, Austria) hematology analyzer with the veterinary software setting for cats.

### 2.4 Cytology

Cytocentrifuge preparations with approximately 5 × 10^5^ cells from the single cell suspensions were stained by a Romanowsky dip stain (LT-Sys Haema-Schnellfaerbung™, Labor+ Technik, Berlin, Germany). All cytologic samples were microscopically inspected by the principal investigator (BR) and a board-certified clinical pathologist (IS). Cellularity, overall preservation of cells and morphology were evaluated. Criteria for cell differentiation included cell size, amount, color, granulation and vacuolization of cytoplasm, and topography, size, shape, and chromatin pattern of the nucleus, as previously described (14).

### 2.5 Flow cytometry

For immunophenotyping aliquots of the single cell suspensions were labelled with anti-feline, anti-human or anti-canine cross-reactive monoclonal antibodies (mAbs) listed in Tables 1, 2. The viability dye eBioscience™ Fixable Viability Dye eFluor™ 780 (Thermo Fischer Scientific, Life Technologies, Carlsbad, CA) was used for live/dead discrimination. Cells only and corresponding isotype controls to all corresponding antibodies were used as controls. All mAbs were directly conjugated to fluorochromes. For each analysis 5 × 10^5^–1 × 10^6^ cells were incubated with the specific mAbs or the isotype controls for 20 min on ice. After a washing step all labelled cells were immediately analyzed in a FACSCanto II flow cytometer™ (BD Biosciences, San Jose, CA, United States). For fixation and permeabilization prior to labelling intracellular antigens, anti CD79acy and anti CD3, the IntraStain-Kit™ (Dako, Glostrup, Denmark) was used according to the manufacturers’ instructions. Combinations of monoclonal antibodies used for multi-color staining and the isotype controls are listed in Table 2.

**Table 1:** List of monoclonal antibodies used for flow cytometric phenotyping of single cell suspension from gastrointestinal feline lymphoma biopsy material including clone, isotype, fluorescent label, source and target species/cross reactivity.

	Clone	Isotype	Fluorescence labelling	Source^a^	Target species / species cross reactivity
CD3	CD3-12	rIgG1	FITC	Bio-Rad	Anti-human (15)
CD4	vpg34	mIgG1	APC	Bio-Rad	Anti-feline
CD5	FE1.1B11	mIgG1	FITC	Bio-Rad	Anti-feline
CD8	vpg9	mIgG1	PE	Bio-Rad	Anti-feline
CD18	CA1.4E9	mIgG1	Alexa647	Bio-Rad	Anti-canine (16)
CD21	CA2.1D6	mIgG1	Alexa647	Bio-Rad	Anti-canine(16, 17)
CD79αcy	HM57	mIgG1	APC	Dako	Anti-human (18)

m, mouse; r, rat; FITC, fluorescein isothiocyanate; APC, allophycocyanin; PE, phycoerythrin.

a Bio-Rad, Hercules, CA, United States; Dako Cytomation, Glostrup, Denmark.

**Table 2:** Antibody combinations used for multi-color flow cytometric phenotyping of feline gastrointestinal lymphoma cells.

Tube Nr.	mAb	Source^a^
1	mIgG1-FITC	Bio-Rad
	mIgG1-PE	Bio-Rad
	mIgG1-Alexa647	Bio-Rad
2	CD4-FITC	Bio-Rad
	CD8-PE	Bio-Rad
	CD18-Alexa647	Bio-Rad
3	mIgG1-FITC	Bio-Rad
	mIgG1-APC	Dako
4	CD5-FITC	Bio-Rad
	CD79-APC	Dako
5	rIgG1-FITC	Bio-Rad
	mIgG1-Alexa647	Bio-Rad
6	CD3-FITC	Bio-Rad
	CD21-Alexa647	Bio-Rad

The tubes with the numbers 1, 3, 5 are the isotype controls followed by the respective specific antibody combinations in tubes 2, 4, 6. All tubes contained exclusively directly conjugated antibodies. Column 1 describes the number of the respective tubes, column 2 the monoclonal antibody, column 3 the company source of the antibody. The cells only and life dead staining control tube is not listed in the Table. In all tubes shown, the eBioscience™ Fixable Viability Dye eFluor™ 780 staining was added. m, mouse; r, rat; FITC, fluorescein isothiocyanate, APC, allophycocyanin; PE, phycoerythrin.

a Bio-Rad, Hercules, CA, United States; Dako Cytomation, Glostrup, Denmark, SouthernBiotech, Birmingham, Alabama, United States.

Gating was performed for all samples using the forward scatter/side scatter (FSC/SSC) dot plot representing the size and the granularity of the cells/events. The target lymphoid population was gated, doublet discrimination was done, and the dead cells excluded by viability stain. The remaining living cells within the gate were used for the analysis of their antigen expression.

As there is no definite proved cut off published for a “monomorphic” expression pattern and a reactive pattern in FCM – in our lab we have the cut off with >90% of the gated cells showing the same expression pattern (eg all CD3^+^CD21^−^ CD4^−^CD8^−^CD18^+^ CD5^−^CD79^−^) – as done in a former publication (12). The only reference available based on feline normal and mildly reactive peripheral lymph nodes describes a range of: CD3^+^ 54.81% ± 11.10%, CD5^+^ 57.39% ± 12.66%, CD21^+^ 40.42% ± 12.40%, CD79αcy^+^ 30.41% ± 13.49%; 30.88% ± 13.48% CD4^+^, 12.91% ± 6.68% CD8^+^ cells (19). Cases eventually presenting with <90% uniformity and subpopulations within the reported ranges were not considered as lymphomas.

### 2.6 Histopathology and immunohistochemistry

The formalin-fixed samples of the gastrointestinal material were embedded in paraffin (FFPE samples), cut in 2 μm sections, stained with haematoxylin and eosin (H&E) and all examined microscopically by two veterinary anatomic pathologists (AFB, TAD).

Immunohistochemistry was performed with a LabVision-Autostainer (Thermo Fisher Scientific, Fremont, United States) using the Bright Vision HRP- Polymer method. FFPE samples were cut in 2 μm sections, deparaffinized, rehydrated and pre-treated with heat in pH 6 citrate for 20 min, incubated in Hydrogen Peroxidase Block (Thermo Fisher Scientific) for 5 min and in Ultra Vision Protein Block (Thermo Fisher Scientific) for another 10 min. A polyclonal rabbit anti-human antibody against CD3 (Dako, Glostrup, Denmark; diluted 1:1000) and a polyclonal rabbit anti-human antibody against CD20 (Spring Biosciences, diluted 1:1000) was used. The samples were incubated with the primary antibodies for 30 min, and subsequently with the secondary antibodies (Bright Vision poly HRP anti rabbit IgG, Immunologic, Duiven, Netherlands) for 30 min. For visualization, DAB Quanto (Thermo Fisher Scientific) for 5 min was used and counterstained with Mayer’s haematoxylin. A feline peripheral lymph node was used as positive control.

All samples were evaluated by one experienced veterinary anatomic pathologist (AFB) and one board certified anatomic pathologist (TAD). Consensus diagnosis was performed using the WHO classification (7).

### 2.7 Lymphocyte clonality testing

For the PCR-based lymphocyte clonality assay, total genomic DNA (gDNA) was extracted from 5 × 10^6^ cells of the feline tissue with 200 μL elution buffer using a commercial kit following the manufacturers’ instructions (E.Z.N.A Tissue DNA Kit™, Omega Biotech, Norcross, Georgia). Genomic DNA concentration and quality were determined using the NanoDrop 2000c™ spectrophotometer (Thermo Fisher Scientific, Waltham, MA, United States) in pedestal mode. The threshold was set to 30 ng/μL with desired 260/280 ratios of 1.8–2.0 and 260/230 ratios above or equal two (2.0–2.2) (20). The gDNA samples were assayed by amplifying a 189 base pair fragment of the feline androgen receptor gene (fAR), the immunoglobulin heavy chain (IGH-VDJ) gene rearrangements with the primer sets V1F2, V3F3 and V3F4 and the T-cell receptor gamma chain (TRG-VJ) gene rearrangements with the primer sets TRG-J1, TRG-J2 and TRG-J3 (20–22). Each PCR reaction was carried out in triplicate including positive and negative PCR controls in each PCR run (20, 23).

After PCR, 10 μL of DNA Dilution Buffer (Qiagen, Hilden, Germany) were added to each PCR reaction and size separated using the QIAxcel Advanced System capillary electrophoresis analyzer with the QIAxcel DNA High Resolution Kit and the QX Alignment Marker 15 bp/1000 bp (Qiagen). The presence and size of obtained PCR products was accurately determined using QIAxcel ScreenGel Software (Qiagen). Identical PCR triplicates verified the reproducibility of the clonality patterns, which were interpreted as described previously (20, 23, 24).

## 3 Results

### 3.1 Patient material

**Figure 1:**
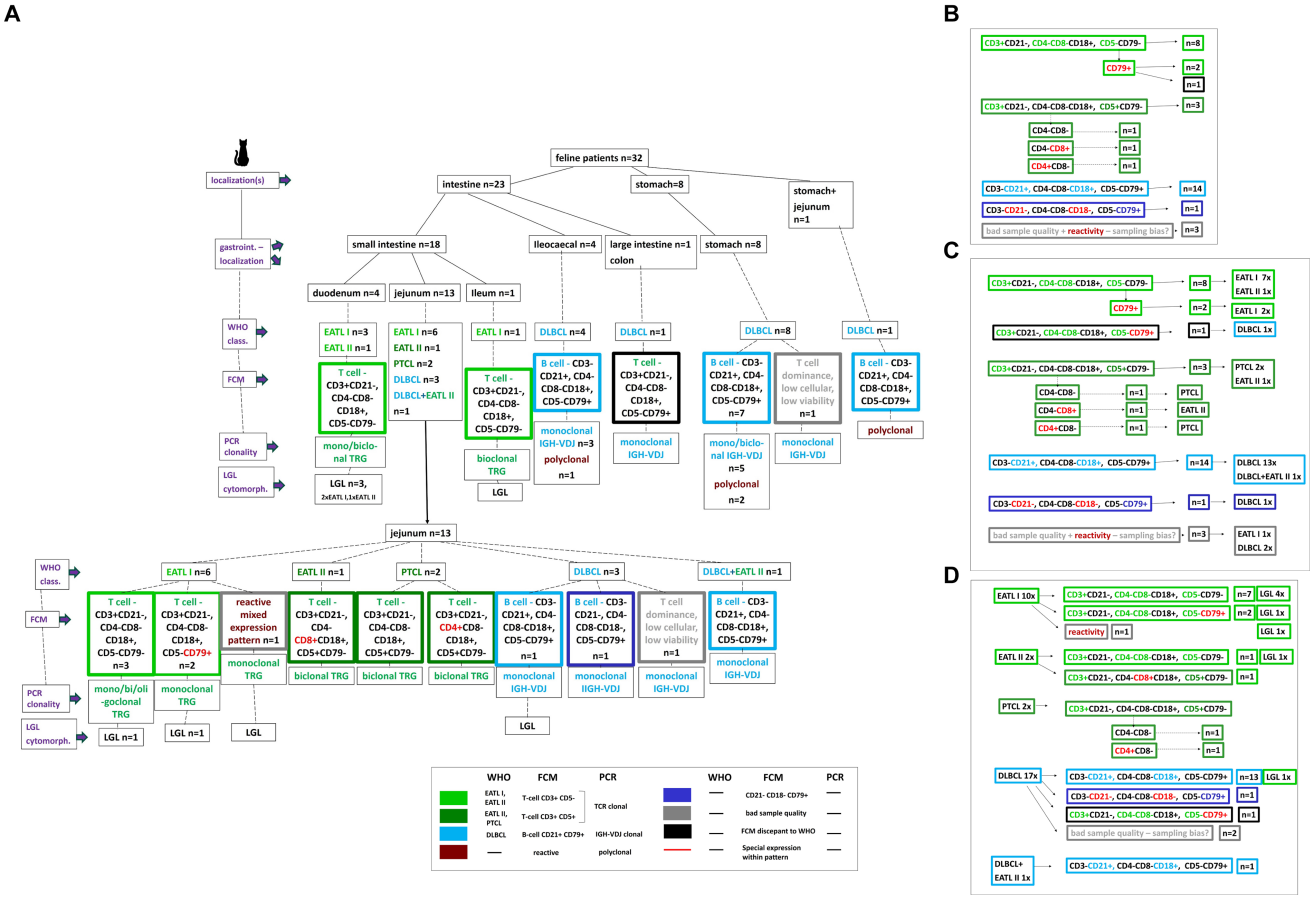
Description of the 32 study patients based on a decision tree. Localizations, specific intestinal localizations, WHO classification, FCM expression patterns, clonality PCR results, LGL cytomorphology are plotted for every single patient **(A)**. A color code clusters the different WHO entities and FCM patterns. Based on the decision tree the results are summarized dependent on the different FCM expression patterns including the respective patient number **(B)**, on the different FCM expression patterns in synopsis with the WHO classification entities including the respective patient number **(C)**, on the WHO classification entities in synopsis with the different FCM expression patterns including the respective patient number **(D)**.

In total, gastrointestinal material from 32 cats with feline lymphoma was collected. Samples originated from 27 (84.5%) domestic shorthair cats, one (3.1%) Maine Coon, one (3.1%) Persian, one (3.1%) Abyssinian, one Norwegian forest cat (3.1%) and one (3.1%) Siamese cat. Ages ranged between 7 months and 14 years (mean 9.1 years, median 9 years). There were 12 (37.5%) castrated males, and 20 (62.5%) spayed females. The 7-month-old patient was FeLV positive. The 3D size of the removed gross gastrointestinal masses was from 3 to 729cm^3^ (mean 84cm^3^, median 20cm^3^). All 32 samples were analyzed by FCM, clonality PCR and histopathology (Figure 1A).

### 3.2 Anatomic site

Eight out of 32 cats (25%) had gastric, 23 (72%) had intestinal lymphoma and 1 (3%) had gastric+jejunal lymphoma. Intestinal lymphoma sites were represented by 18 small intestinal, 4 ileocaecal, 1 large intestinal.

The 18 small intestinal lymphoma sites were 4 duodenum, 13 jejunum, one ileum (Figure 1A).

### 3.3 Total nucleated cell count of single cell suspension for FCM

Numbers of cells harvested from sampled gastrointestinal material of 27/32 patients ranged from 0.26×10^7^ to 49×10^7^ cells (mean 17.8×10^7^, median 17.4 ×10^7^). The gated population for the FCM analyses was from 11.3 to 99% (mean 39%, median 41.8%), cells alive were ranging from 38 to 99% (mean 87%, median 71%).

### 3.4 Cytology

**Figure 2:**
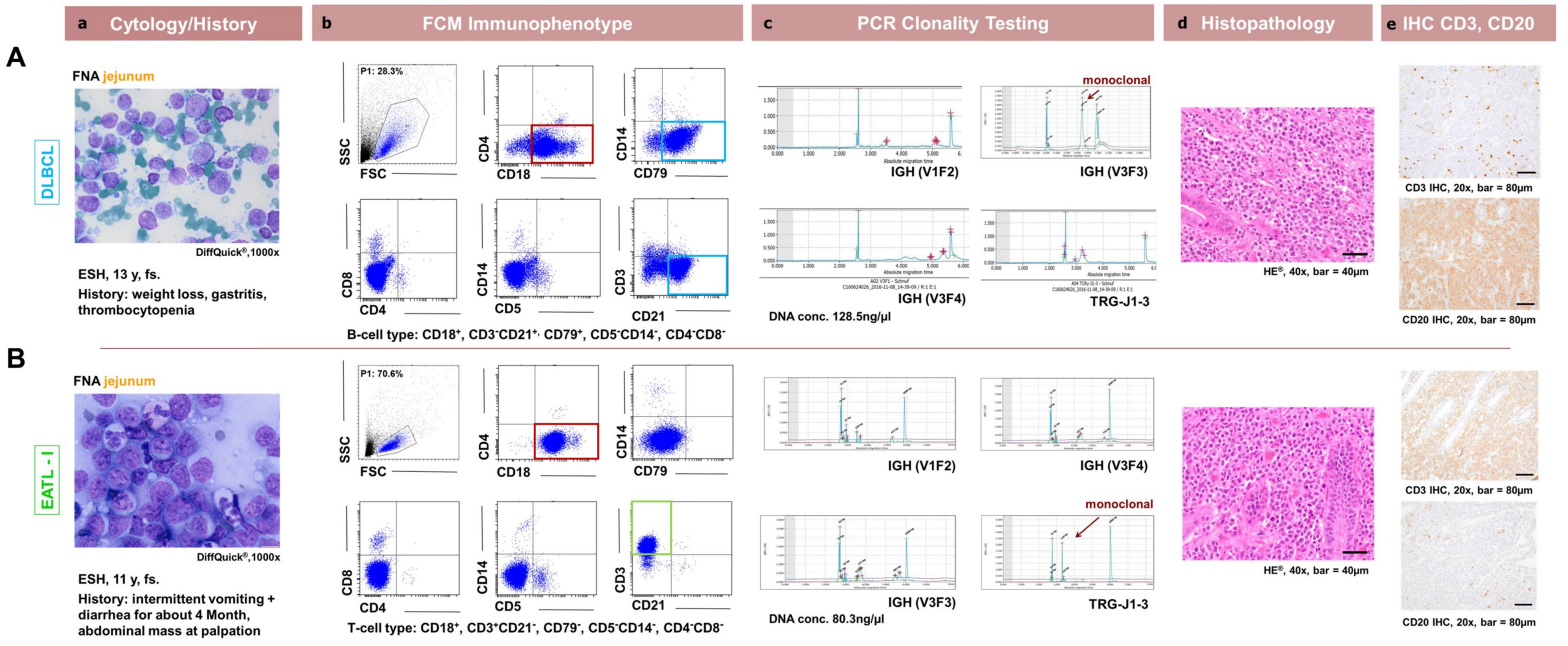
Comparison between patient cytology, flow cytometry, PCR for lymphocyte clonality testing, histopathology and immunohistochemistry in feline intestinal lymphoma immunophenotyping. Corresponding cytological (**a**, DiffQuick^®^1,000x), flow cytometrical plots for Immunophenotyping (**b**, showing the corresponding FSC/SSC (P1), CD18/CD4, CD79/SSC, CD4/CD8, CD5/SSC, CD21/CD3 dot plots), (**c**, capillary electropherogram traces for IGH-VDJ and TRG (V1F1, V3F3, V3F4, TRG-J1-3) including the DNA concentration in ng/μl evaluated photometrically) clonality PCR traces, histopathological (**d**, H + E, 400x) and immunohistochemical (**e**, DAB chromogen, 200x) images are shown for 2 representative patients. In the first row **(A)** represents a 13y, fs ESH cat with a jejunal DLBCL showing CD3^−^ **CD21^+^** CD4^−^CD8^−^ **CD18^+^** CD5^−^ **CD79^+^** expression and a monoclonal IGH-VDJ antigen receptor rearrangement and CD20+ with IHC. In the second row **(B)** represents a 11y, fs ESH cat with a jejunal EATLI showing **CD3^+^** CD21^−^ CD4^−^CD8^−^ **CD18^+^** CD5^−^CD79^−^ expression and a monoclonal TRG antigen receptor rearrangement and CD3+ with IHC. The FSC/SSC (P1) gated population is marked in blue **(Ab,Bb)** and is represented in the subsequent dot plots. It is chosen due to size and granularity and life / dead discrimination. The cytological images are consistent with lymphoma for each case. CD18 expression is marked in red, CD3 expression in green and CD21 and CD79 expression in light blue.

The cytomorphologic evaluation of the gastrointestinal material was performed in all 32 cases as part of inclusion into the study using FNA material and was repeated with the solid material from the surgically removed mass with cytospins and impression smears. Cytology was consistent with lymphoma in all cases. All samples showed intermediate to large size lymphoid cells with immature chromatin (Figures 2Aa+b). Morphologically inconclusive needing additional confirmation with clonality PCR prior to further diagnostics or therapy were not included. Large granulocyte lymphocyte (LGL) lymphoma was observed in 8 patients. Seven out of these eight were T-cell lymphomas. Three were in the duodenum, 4 in the jejunum and one in the ileum (Figures 1A+D).

### 3.5 Immunophenotyping – flow cytometry

#### 3.5.1 Immunophenotypic expression patterns

##### 3.5.1.1 T-cell immunophenotype

The most common T-cell phenotype was **CD3**^**+**^CD21^−^ **CD4**^**−**^**CD8**^**−**^**CD18**^**+**^ CD5^−^CD79^−^ in 8 cases. Three cases were **CD3**^**+**^CD21^−^ **CD4**^**−**^**CD8**^**−**^**CD18**^**+**^ CD5^−^**CD79**^**+**^. Three cases were positive for CD3 and CD5 but showing a variable CD4CD8 expression. One case each of these were **CD3**^**+**^CD21^−^ **CD4**^**−**^**CD8**^**−**^**CD18**^**+**^ **CD5**^**+**^CD79^−^, **CD3**^**+**^CD21^−^ **CD4**^**+**^CD8^−^**CD18**^**+**^ CD5^−^CD79^−^ and **CD3**^**+**^CD21^−^ CD4^−^**CD8**^**+**^**CD18**^**+**^ CD5^−^CD79^−^ (Figure 1B).

##### 3.5.1.2 B-cell immunophenotype

The dominating B-cell immunophenotype was CD3^−^**CD21**^**+**^ CD4^−^CD8^−^**CD18**^**+**^ CD5^−^**CD79**^**+**^ in 14 cases. One was CD3^−^CD21^−^ CD4^−^CD8^−^CD18^−^ CD5^−^**CD79**^**+**^.

Three cases did not show a definitive immunophenotypic pattern. Two were of T cell dominance with <10% B cells; most suggestive but not definitive of T cell lymphoma and one showed mixed lymphoid cells compatible with a reactive phenotype expression pattern.

From the values here these expression results of these patients were between published ranges and < 90% uniformity (12) – so they were not considered as definitive lymphomas in FCM.

#### 3.5.2 Immunophenotypic expression based on localization

The most common small intestinal T-cell phenotype was **CD3**^**+**^CD21^−^ **CD4**^**−**^**CD8**^**−**^**CD18**^**+**^ CD5^−^CD79^−^ (Figures 1A, 2Bb) in 8/18 cases. Four are localized in the duodenum, 3 in the jejunum and one in the ileum. Two cases showed the same expression pattern except of being **CD79+**. All these cases were CD3+ and CD5- and were localized in the jejunum. In contrast to this, 3 jejunal cases were positive for CD3 and CD5 but showing a variable CD4CD8 expression. One case each of these were **CD3**^**+**^CD21^−^ **CD4**^**−**^**CD8**^**−**^**CD18**^**+**^ **CD5**^**+**^CD79^−^, **CD3**^**+**^CD21^−^ **CD4**^**+**^CD8^−^**CD18**^**+**^ CD5^−^CD79^−^ and **CD3**^**+**^CD21^−^ CD4^−^**CD8**^**+**^**CD18**^**+**^ CD5^−^CD79^−^. Of the remaining small intestinal samples, 2 showed a CD3^−^**CD21**^**+**^ CD4^−^CD8^−^**CD18**^**+**^ CD5^−^**CD79**^**+**^ B-cell phenotype, one was negative for all antibodies except CD79 (CD3^−^CD21^−^ CD4^−^CD8^−^CD18^−^ CD5^−^**CD79**^**+**^), one showed a mixed lymphoid cell expression compatible with a reactive flow cytometric immunophenotype and one T cell dominance with <10% B cells; most suggestive but not definitve of T cell lymphoma. These were all located in the jejunum (Figure 1A).

All 4 lymphomas located ileocaecal had the same expression B-cell pattern of CD3^−^**CD21**^**+**^ CD4^−^CD8^−^**CD18**^**+**^ CD5^−^**CD79**^**+**^ (Figures 1A, 2Ba).

Seven out of the 8 gastric lymphomas the same B-cell of CD3^−^**CD21**^**+**^ CD4^−^CD8^−^**CD18**^**+**^ CD5^−^**CD79**^**+**^ as well. One gastric lymphoma was of T cell dominance with <10% B cells; most suggestive but not definitive of T cell lymphoma (Figure 1A).

The one large intestinal lymphoma was of T-cell expression being **CD3**^**+**^CD21^−^ **CD4**^**−**^**CD8**^**−**^**CD18**^**+**^ CD5^−^**CD79**^**+**^ (Figure 1A).

The last remaining case localized in stomach/jejunum was of the dominant B-cell pattern of CD3^−^**CD21**^**+**^ CD4^−^CD8^−^**CD18**^**+**^ CD5^−^**CD79**^**+**^ (Figure 1A).

### 3.6 Histopathology and immunohistochemistry based on localization

Small intestinal lymphomas were represented by 10 EATL I, 2 EATL II, 2 PTCL, 3 DLBCL and one DLBCL+EATL II. Within the small intestinal lymphomas, the 4 duodenal lymphomas were 3 EATL I and 1 EATL II. The 13 jejunal lymphomas were 6 EATL I, 1 EATL II, 2 PTCL, 3 DLBCL and one DLBCL+EATL II. All 8 gastric lymphomas were DLBCL.

The 4 ileocaecal lymphomas were all DLBCL. Also, the one large intestinal lymphoma (colon) was a DLBCL as well as the lymphoma located in stomach and jejunum (Figures 1A, 2d+e).

Abdominal lymph node involvement was observed by histopathology in 12/17 DLBCL, 2/2 PTCL, 3/10 EATL I, 1/2 EATL II and in the DLBCL+EATL II case.

### 3.7 Lymphocyte clonality testing

Clonality testing, performed in all 32 patients, showed clonality for TRG in 14 cases, with IGH-VDJ clonality in 14 samples. A polyclonal result was seen in 4 B-cell lymphoma cases. Out of the TRG clonal PCR results, 8 were monoclonal, 5 were biclonal and one was oligoclonal. The clonal IGH-V DJ cases were represented by 13 monoclonal and one biclonal result (Figures 1A, 2c).

In summary clonality PCR results were positive in 87.5% (28/32) of all cases. No cross-lineage rearrangement was observed.

### 3.8 Immunophenotypic expression matched with localization, histopathologic who classification and clonality PCR

The most common small intestinal T-cell phenotype was **CD3**^**+**^CD21^−^ **CD4**^**−**^**CD8**^**−**^CD18^+^ **CD5**^**−**^**CD79**^**−**^ seen in 8 cases. Seven out of these were EATL I and one was EATL II in all small intestinal areas.

Three cases were **CD3**^**+**^CD21^−^ **CD4**^**−**^**CD8**^**−**^CD18^+^ **CD5**^**−**^**CD79**^**+**^. Two of them were EATL I in the jejunum, one was a DLBCL in the colon.

The three cases being **CD5**^**+**^ in contrast to the before mentioned samples were of variable CD4CD8 expression. The CD4^−^**CD8**^**+**^ cytotoxic T-cell lymphoma was jejunal EATL II, the **CD4**^**+**^CD8^−^ T-helper cell lymphoma a jejunal PTCL and the **CD4**^**−**^**CD8**^**−**^ lymphoma a jejunal PTCL as well.

The dominating expression pattern in the 14 B-cell lymphomas, CD3^−^**CD21**^**+**^ CD4^−^CD8^−^CD18^+^ CD5^−^**CD79**^**+**^ was in all except one case diagnosed as DLBCL. Localization was ileocaecal (4), stomach (7), stomach+jejunum (1), and jejunum (1). One case was histopathologically a jejunal DLBCL+EATL II.

One jejunal DLBCL lymphoma case was CD3^−^**CD21**^**−**^ CD4^−^CD8^−^CD18^−^ CD5^−^**CD79**^**+**^.

One jejunal case showed heterogeneous lymphoid cells compatible with reactive immunophenotype being diagnosed as EATL I. The two cases showing a dominant T-cell expression with <10% B cells were both diagnosed with DLBCL in histopathology. All patterns see Figures 1C +D.

A polyclonal clonality PCR result was seen in 4/32 cases representing a false negative result based on FCM and histopathology (Figure 1A). These were all DLBCL lymphoma. All other TRG and IGH-VDJ clonality results were in all cases in line with histopathology and immunohistochemistry.

IHC and FCM results were in agreement in 87.5% (28/32) of all cases. When all 3 methods were combined, consistent results were seen in 75% (24/32).

## 4 Discussion

This is the first report of FCM data obtained by a standardized multi-color approach aligned to WHO classification and PCR-clonality testing in cats with GI-lymphoma.

In this cohort the CD4^−^CD8^−^ immunophenotype predominated in both most frequently affected small intestinal sites (duodenum and jejunum). One study investigated CD4CD8 in feline low grade intestinal T-cell lymphoma (LGITL) using IHC of frozen samples in 11 cats, predominantly with jejunal LGITL (3). Four out of 11 (36%) cases were CD4^−^CD8^−^, four (36%) were CD4^+^CD8^−^ and three cases (27%) were CD4^−^CD8^+^. The more homogeneous distribution between CD4 and CD8 positivity in the latter report might be explained by the different WHO entities investigated in our dataset namely EATL I and EATL II. Nevertheless, in our cohort CD3^+^CD4^−^CD8^−^CD5^−^predominated in EATL I.

Regarding the CD4CD8 expression in physiologic feline intestinal epithelial lymphocytes (IELs) Roccabianca et al. (11) reported a high prevalence (44%) of CD4^−^CD8^−^ IELs in specific pathogen free (SPF) healthy cats. In other species like the dog, comparable CD4^−^CD8^−^ IEL cell populations was reported only in neonatal healthy Swiss Beagle dogs showing TCRɣδ^+^ and representing approximately 20% of all IELs. However, the dominant remaining (80%) TCRαβ^+^ IELs in these neonatal and adult Beagles did not comprise more than 10% CD4^−^CD8^−^ cells (25). This fact leads to the conclusion that in the canine species a population exceeding 10% of CD4^−^CD8^−^ TCRαβ^+^ IELs is considered atypical. To the authors knowledge there is no anti- TCRαβ/ɣδ available for FCM in cats so that a full comparison between species is currently not possible. However, cats may contain a unique IEL phenotype as described above in Roccabianca et al. (11) in IELs in SPF healthy cats regarding their CD4CD8 expression. For this reason, a predominance of this double negative population in feline intestinal FCM might indicate neoplastic disease, if confirmed by a larger body of evidence in the future. Until then clonality testing is a helpful complementary tool, because immunophenotypic dominance cannot discriminate between a clonal versus polyclonal population. In the present study all CD4^−^CD8^−^ cases showed a clonal result. This fact emphasizes the necessity of a multimodal diagnostic approach and underlines the usefulness of clonality testing as an adjacent tool discriminating between reactive and malignant lymphocyte populations.

In our case series only one **CD3**^**+**^CD21^−^ **CD4**^**+**^CD8^−^**CD18**^**+**^ CD5^−^CD79^−^ helper cell lymphoma and one **CD3**^**+**^CD21^−^ CD4^−^**CD8**^**+**^**CD18**^**+**^ CD5^−^CD79^−^ cytotoxic T cell lymphoma was observed. The cytotoxic T-cell lymphoma was EATL II and the T-helper cell lymphoma a PTCL.

In the study by Freiche and colleagues 36% of LGITL were CD4^+^ and 27% were CD8^+^ (3). Again, this discrepancy is most likely caused by the different WHO entities investigated and needs further studies with an extended FCM panel including CD4 and CD8.

Nevertheless, all cases in our series showing either CD4 or CD8 positivity also expressed CD5. In contrast, all CD4^−^CD8^−^ negative cases except one were also negative for CD5. In addition to that, no EATL I lymphoma showed CD4, CD8 nor CD5 positivity. All CD5 positive cases were represented by EATL II or PTCL. This finding needs further investigation because it might indicate a relation between immunophenotype and WHO entities.

Three intestinal cases (21.4%) showed double positive expression for B and T-cell antigens being **CD3**^**+**^CD21^−^ CD4^−^CD8^−^**CD18**^**+**^ CD5^−^**CD79**^**+**^. Two of them were EATL I, and one a DLBCL.

In recent years, many groups performing diagnostic FCM in the veterinary field observed non-specific binding of neoplastic T cells to CD79αcy (W Vernau, 2021, personal communication). In dogs and in cats, non-specific binding of neoplastic T cells has been observed. Thus, CD79αcy must be considered a less reliable antibody for labelling B cells than CD21 (19). The single case of DLBCL exhibiting CD3 positivity in our case series was the only one showing discrepant results with histopathology and clonality testing being monoclonal for IGH-VDJ.

In general, CD3 is considered a reliable antibody against T-cells in many species, including domestic cats. However, an aberrant expression in this case cannot be excluded. In humans a false positive CD3 expression is reported to be related with 24–72 h storage (26). This can be excluded in our case because all samples were processed within 1 hour after surgery.

When all 3 methods, FCM, WHO classification and clonality PCR were combined, consistent results were seen in 75% (24/32). One of the 8 discrepant results is the CD3^+^ CD79^+^ case mentioned above for which an explanation for the FCM mismatch could not be identified. For the remaining 7 discrepant results reasonable explanations were found. Three cases showed low cellularity and cell viability in FCM so that heterogeneous lymphoid cells compatible with a reactive phenotype and T-cell predominance was observed. Two of them were DLBCL, one in the stomach, one in the jejunum and the remaining case was a jejunal EATL I with consistency between WHO and clonality PCR. A preanalytical problem in processing the sample is the most likely explanation. These cases are reported to underline the fact that a multimodal approach is advisable especially when clinical findings (tumor size!) indicate neoplasia.

A probably false negative PCR result is assumed in the remaining inconsistent cases as the results are overruled by histopathology as current gold standard. The false negative rate for clonality PCR was 12.5% resulting in a diagnostic sensitivity of 87.5% which is slightly below the expected sensitivity of 90% for this assay (23). All false negative cases were DLBCL lymphomas. Somatic hypermutations which can occur with B-cell neoplasia might be a possible explanation (24). All other TRG and IGH-VDJ clonality results agreed with immunophenotyping using histopathology and immunohistochemistry, thus no cross-lineage rearrangement was observed.

Regarding the B-cell immunophenotyping, the dominant expression pattern in the 14 B-cell lymphomas was CD3^−^**CD21**^**+**^ CD4^−^CD8^−^**CD18**^**+**^ CD5^−^**CD79**^**+**^ in the 7 stomach, all 4 ileocaecal region, 1 jejunal and the one stomach+jejunum cases. These were all DLBCL. This agrees with FCM expression patterns for DLBCL in other species (27).

One case showing the same expression as above, CD3^−^**CD21**^**+**^ CD4^−^CD8^−^**CD18**^**+**^ CD5^−^**CD79**^**+**^, comprised two separate concurrent histopathologic populations; a small cell mucosal lymphoma (EATLII), superimposed by a DLBCL, confirmed with IHC. This entity is not described in the WHO but observed occasionally in feline intestinal lymphoma cases (A Durham, 2022, personal communication). In FCM and clonality PCR testing, the T-cell lymphoma population was not detected. Sampling bias for FCM and clonality PCR is the most likely explanation.

One jejunal DLBCL lymphoma case was CD3^−^CD21^−^ CD4^−^CD8^−^CD18^−^ CD5^−^**CD79**^**+**^. The solitary positivity for CD79 with negativity for the pan antibody CD18 and the reliable B-cell CD21 makes this FCM result not reliable for a B-cell type. However, DLBCL diagnosis supported by monoclonality for IGH-VDJ were proof for B-cells in this case. The loss of CD18 positivity in a B-cell lymphoma has not been described so far. This should be investigated in the future. This underlines in addition inclusion of further test in such cases.

Further studies using an extended panel of antibodies in a multi-color FCM approach are needed for better understanding of different expression patterns. Nevertheless, in the preset cohort, the frequent FCM B-cell immunophenotype in 13/14 B-cell cases seems to be more uniformly expressed within the DLBCL entity even in different sites along the GI-tract. In contrast to this, more diverse expression patterns are seen in the T-cell immunophenotype throughout the different small intestinal regions.

In general, the distribution of B and T-cell phenotype with dominance of T-cell immunophenotypic EATL I in the small intestine and B-cell FCM immunophenotypic DLBCL in gastic, ileocaecal and large intestine (colon) agrees with former reports (5).

## 5 Conclusion

We describe different FCM expression patterns of WHO entities in feline GI-lymphoma of different sites aligned with PCRclonality results in a cohort of 32 patients. Results are intended to provide a starting point for further research attempting to match FCM expression patterns to WHO-entities as already done in other species (28). Until more data are available a multimodal approach is still recommended.

## 6 Limitations of the study

A low and heterogeneous case load of 32 patients is the first limitation, which prohibited in depth statistical workup. The other limitation is the inclusion of discrepant results in some cases, which raise more questions than answers. We decided to report these findings to share our experience and stimulate further discussions.

## Data availability statement

The raw data supporting the conclusions of this article will be made available by the authors, without undue reservation.

## Ethics statement

The animal studies were approved by Ethics Committee and the Federal Ministry for Science and Research (referring number: GZ 68.205/0173-WF/V/3b/2015). The studies were conducted in accordance with the local legislation and institutional requirements. Written informed consent was obtained from the owners for the participation of their animals in this study.

## Author contributions

BR: Conceptualization, Data curation, Formal analysis, Methodology, Visualization, Writing – original draft. BW: Project administration, Supervision, Writing – review & editing. DB: Formal analysis, Writing – review & editing. SH: Writing – review & editing. SG: Writing – review & editing. KH: Writing – review & editing. GG: Writing – review & editing. AF-B: Writing – review & editing. TD: Writing – review & editing. IS: Writing – review & editing.
